# A Case of Skin Grafting

**Published:** 1876-02

**Authors:** B. M. Cromwell

**Affiliations:** South Georgia Medical Society, Albany, Ga.


ATLANTA

MEDICAL AND SURGICAL JOURNAL.

VOL. XIII.] FEBRUARY—1876. [No. 11.

### Original Communications.

### A CASE OF SKIN GRAFTING, WITH COMMENTS.

BY B. M. CROMWELL, M.D.

*Read before the South Georgia Medical Society, Albany, Ga., Dec. 14, 1875.*

Spencer Daniel, (col.) æt. about fifty-two years, was kicked by a horse on the shin, four years ago, which resulted in an ulcer. Is strong and healthy, having about him no marks of constitutional disease. Received treatment at various times, but without being cured. When he applied to me in August, the ulcer was all of eight inches in its longest diameter, and about five and a half or six inches wide. The tibia was exposed and necrosed for five inches, the necrosed bone being movable and ready to come away. He was directed to return to his home (in the country) and arrange his affairs so that he could be under observation and treatment for at least three months; for I would not undertake his treatment for a less period.

He accordingly presented himself at the County Hospital, and his leg was first dressed on October 15th. The necrosed bone had in the meantime come away. I show you here the piece; it is five inches long and from one-eighth to one-quarter of an inch thick. The dressing for the following week consisted of an ointment of balsam of peru on lint, and over this a well-applied roller bandage from the toes to the knee. I should have mentioned that the ulcer was in every respect healthy on admission, and remained so; its length at this time was about seven inches and its width four and a half or five inches.

October 20th.—Made thirteen transplantations of skin into the ulcer, which continues healthy and in a most promising condition for the success of the operation. The pieces of implanted skin were about the size of canary seed, others a little larger, about the size of barley corns. The grafts were taken from sound skin on the upper part of the same leg, and were made by hooking up a little point of skin with a tenaculum, and snipping it off with a pair of scissors. The grafts were then placed upon the ulcer (cut surface down) at intervals of about an inch each way, and were gently pressed against the granulating surface with the curve of the tenaculum until all air was expelled. After they were all applied, a piece of lint smeared with the Peruvian ointment was carefully applied to the ulcer; the leg was then bandaged and the dressing allowed to remain two days before being disturbed.

October 22d.—The bandage being removed, the lint was well soaked in warm water, and then carefully peeled off, care being taken not to disturb the grafts; after which, the ulcer being cleansed of the pus upon it by allowing a stream of water to trickle over it, the grafts were examined. Eight were found adherent, and five came off with the dressing. The dressing and bandage were re-applied every two days. At each dressing the progress of the grafts was watched with great interest. For the first few days no perceptible change could be observed; the grafts appeared to be merely sticking to the surface of the ulcer, like any foreign matter would; but after a while a change in the appearance of the ulcer around the margin of the grafts became apparent. The granulations seemed to have receded for an appreciable space, and the intervening space was smooth and more vascular. The little grafts about this time lost their cuticle, so that to the casual observer the whole thing looked like an abortion. Very soon, however, each graft formed a new cuticle of its own, which, beginning in the centre as a mere speck, radiated in all directions, the graft growing by proliferation of its own cells.

November 3d.—Ten other grafts were added to those already transplanted, four of which lived and pursued the same course as the others. At this time the original ones were about the size of dimes; one had coalesced with the margin of the ulcer, and several others were in close proximity.

November 12th.—Size of grafts was from five-eighths to seven-eighths of an inch in diameter; length of ulcer six inches, width three and one-half inches. Inserted five new grafts along the outer margin of the ulcer and close to it, and one at its lower extremity. These grafts were from two to four times larger than any that had been put in. They were taken from the thigh of the opposite side, by pinching up pieces of skin with a pair of forceps, and cutting them off with a sharp bistoury, making grafts about the size of a gold dollar or somewhat smaller. They were strapped to the ulcer by strips of isinglass plaster, and the dressing and bandage applied as before.

November 14th.—On removing the dressing the grafts appeared to be doing well; strips of plaster not disturbed.

November 16.—On renewing the dressing and strips to-day with less caution than usual, one of the grafts was torn from its bed, and bled freely, showing that circulation had been established in it, and that union had taken place. The others were adherent, but showed as yet no signs of growth. I find two of the original grafts have united with the margin, and one with two of those planted November 3.

Instead of the usual dressing, applied Cowen's "Ulcer Food," which consists of wheat flour oz. 4, pulv. acacia, pulv. magacinth., each oz. $\frac{1}{2}$; chalk grs. 2, and one egg, to be boiled for one minute, and when cool to be applied three or four times a day to the ulcer with a soft brush. The object of this application was to see if the ulcer could not be stimulated to an extraordinarily rapid healing, which would more than compensate for the constrained position (leg in afracture-box) that had to be kept by the patient. The results, however, did not warrant its continued use. For the five days the paste was applied the ulcer gained by its margin and by the grafts, but not more, I thought, than it did by the usual dressing; it was discontinued, therefore, and the balsam and bandage reapplied.

It is unnecessary to continue the detailed history of the case. The ulcer continued to contract from its margin and by the growth of the grafts without interruption, until the result I show you to-day (Dec. 14) has been attained. (The grafts were shown to have united in all directions with each other and the margin, leaving a serpegenous line of unhealed tissue, that equalled about a square inch in all, or perhaps a little more. The black points marking the original grafts, gradually enlarging, showed well on the light background of the recently healed ulcer, each one of which could be seen and counted.)

The practice of skin grafting was first introduced in Paris, by

M. Reverden, in the year 1869, and was speedily introduced into the London hospitals by Mr, Polock and others, with such uniform and gratifying success, that in a short time the operation became the established mode of procedure with a certain class of ulcers in the metropolitan hospitals. In this country the practice has enjoyed the same popularity with surgeons of hospitals as in Europe; but from the sources of information at my command, I cannot say that it has received that attention at the hands of general practitioners throughout the country that I think its merits entitle it to.

In presenting this case, therefore, to the Society, I have dwelt at some length upon details that may prove wearisome; but convinced, as I am, that the treatment pursued opens up a better and more hopeful prospect for the successful management of these disheartening cases than any heretofore employed, I thought it best to give such details as would furnish those who wish to try it some data to go upon.

To the general practitioner, an old ulcerated leg is the very *bete noir* of his profession; his spirit dies within him when one presents itself, and if, with a generous donation of "salve" he can be rid of his patient and quiet his conscience, he rejoices at the thought of how much time, annoyance, and ultimate disappointment have been spared him. Indeed, such cases cannot be otherwise than disappointing; for besides the fact that the class which is most troubled with sore legs, is the least able to remunerate a physician for the great amount of very disagreeable work he has to do in treating an ulcer, such persons are least able to spare the time from their daily avocations to get well. With them time is literally money, and unless they go upon the county (a thing they are generally loth to do) that rest cannot be given to the ulcer, without which all plans of treatment, except the one under consideration, must fail.

There are limits beyond which an ulcer will not heal. The reparative powers of nature seem to become exhausted in the effort, and after reaching a certain point they cease. Who has not had such cases, and waited patiently on such ulcers for months, only at last to find that it had done all it was going to do, and would heal no more?

Now these skin grafts are great economists of time in treating an ulcer, as this case shows you; and if that was all they accomplished their aid would be invaluable. But the uniform testimony of those who have practised the method is, that their presence in the ulcer stimulates it to greater activity, and that cicatrization proceeds from the margin much more rapidly than before their introduction. Besides these good results, the cicatrices of which they form a part are not so tender or liable to be abraded or broken down. In extensive burns or scalds, where the resulting cicatrization would contract the skin and produce those ugly deformities that are so familiar to us, the application of numerous grafts would tend to fill the ulcer up with skin taken from other parts of the body, and these unsightly and painful deformities would be prevented or much modified. Hence there is not an aspect from which the operation is viewed that is not highly gratifying. The patient becomes interested in what is going on, and is encouraged to persevere in a system that entails severe restraint and self-denial which gives such palpable results, and the physician is rewarded in part by the interest he takes in this novel and interesting experiment, as well as in the thought that he is putting a fellow-man on his pins again, and so enabling him to enter again life's arena less heavily weighted in the race.

The size of the grafts usually taken varies from that of a canary seed to that of a barley corn. In some instances larger ones are used, but the pain they give in cutting them out, and the difficulty of thoroughly freeing them of all cellular substance etc., makes the above sizes preferable. They may be taken from any locality, the arms, the thigh, chest or back. Those I put in (the first) were taken from the leg on which the ulcer was, but from a part that had never been diseased. They may be taken also from another person, or from a freshly amputated limb, when the amputation was for an accident, and not for disease; one instance being mentioned where the graft (a large one) was taken from a negro, the recipient being white. It grew beautifully, and never changed its character or its color.

The plan usually pursued is to pinch up a minute portion of skin with a pair of forceps, and to cut it off with a sharp bistoury, lancet or pair of scissors. The graft is then placed on the granulating surface and gently pressed on it; after all the grafts have been placed in position, they are kept in place by strips of isinglass plaster, which are not to be removed for several days (three or four). Great care should be observed in removing the first dressings, for the grafts have but a feeble hold for the first week, and are easily torn from their bed.

The great pre-requisites for the success of this operation are that the patient should be in good health (not robust necessarily, but free from acute disease, and healthy in the performance of all his functions), and that the ulcer should be clean and granulating healthily. Yet it is proper to remark that flabby and ill-conditioned ulcers have been known to take the grafts, and that callous ulcers are those, according to some authors, that are most benefited by the operation. Even ulcers on persons tainted with syphilis form no exception to the benefits to be derived, but in such cases constitutional must accompany local treatment.

